# Prevention, diagnosis, and treatment protocol of dengue during pregnancy and the postpartum period

**DOI:** 10.61622/rbgo/2024rbgo73

**Published:** 2024-06-27

**Authors:** Geraldo Duarte, Antonio Rodrigues Braga, Regis Kreitchmann, Maria Luiza Bezerra Menezes, Angélica Espinosa Barbosa Miranda, Ana Gabriela Alvares Travassos, Patrícia Pereira dos Santos Melli, Roseli Mieko Yamamoto Nomura, Agnaldo Lopes da Silva, Maria Celeste Osório Wender

**Affiliations:** 1 Faculdade de Medicina de Ribeirão Preto Universidade de São Paulo Ribeirão Preto SP Brazil Faculdade de Medicina de Ribeirão Preto, Universidade de São Paulo, Ribeirão Preto, SP, Brazil.; 2 Faculdade de Medicina Universidade Federal do Rio de Janeiro Rio de Janeiro RJ Brazil Faculdade de Medicina, Universidade Federal do Rio de Janeiro, Rio de Janeiro, RJ, Brazil.; 3 Universidade Federal de Ciências da Saúde de Porto Alegre Porto Alegre RS Brazil Universidade Federal de Ciências da Saúde de Porto Alegre, Porto Alegre, RS, Brazil.; 4 Faculdade de Ciências Médicas Universidade de Pernambuco Recife PE Brazil Faculdade de Ciências Médicas, Universidade de Pernambuco, Recife, PE, Brazil.; 5 Faculdade de Medicina Universidade Federal do Espírito Santo Vitória ES Brazil Faculdade de Medicina, Universidade Federal do Espírito Santo, Vitória, ES, Brazil.; 6 Faculdade de Medicina Universidade do Estado da Bahia Salvador BA Brazil Faculdade de Medicina, Universidade do Estado da Bahia, Salvador, BA, Brazil.; 7 Hospital das Clínicas Faculdade de Medicina de Ribeirão Preto Universidade de São Paulo Ribeirão Preto SP Brazil Hospital das Clínicas, Faculdade de Medicina de Ribeirão Preto, Universidade de São Paulo, Ribeirão Preto, SP, Brazil.; 8 Escola Paulista de Medicina Universidade Federal de São Paulo São Paulo SP Brazil Escola Paulista de Medicina, Universidade Federal de São Paulo, São Paulo, SP, Brazil.; 9 Faculdade de Medicina Universidade de São Paulo São Paulo SP Brazil Faculdade de Medicina, Universidade de São Paulo, São Paulo, SP, Brazil.; 10 Universidade Federal de Minas Gerais Belo Horizonte MG Brazil Universidade Federal de Minas Gerais, Belo Horizonte, MG, Brazil.; 11 Universidade Federal do Rio Grande do Sul Porto Alegre RS Brazil Universidade Federal do Rio Grande do Sul, Porto Alegre, RS, Brazil.

## Introduction

Over the last few decades, Brazil has faced significant challenges related to infection by the dengue virus (DENV) transmitted by the *Aedes aegypti* mosquito. Dengue is endemic in many tropical and subtropical regions of the planet, often leading to epidemic outbreaks of varying intensity and frequency, influenced by factors such as rising temperatures, catastrophic rainfall, humidity, disorderly urbanization and lack of adequate sanitation, among other causes.^(1,2,3)^ The Brazilian government has implemented several strategies over the years to control the spread of DENV, including public awareness campaigns, vector control programs, environmental management measures and more recently, the vaccine.^(4)^ However, as a consequence of the complexity of the life cycle of the mosquito vector and poor basic sanitation in some areas, the challenges persist. The adaptive capacity of *Aedes aegypti* has also significantly reduced the effectiveness of control strategies such as the use of insecticides, demanding constant changes. In this scenario, the DENV infection continues to challenge public health services, presenting high rates of morbidity, and mortality and confirming that little real progress towards its control has actually been achieved.^(5)^

**Figure 1:**
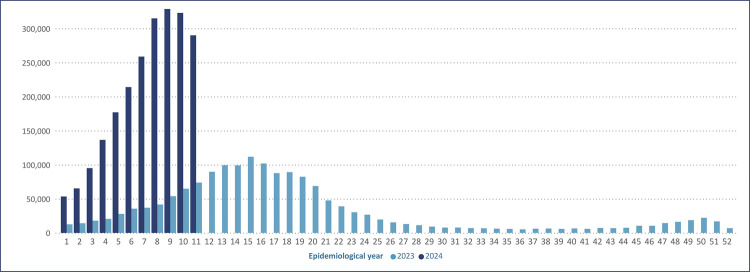
Number of probable dengue cases per epidemiological week (2023 and 2024)

Source: SINAN. Brazil (2024).^(6,7)^

Dengue data in Brazil for the period of epidemiological weeks 01-11 of 2024 compared to the same period in 2023 demonstrate a significant increase in incidence in 2024, with effective anticipation of the increase in the number of cases (Figure 1). The weekly variation in the number of dengue cases in the country can be accessed at https://www.gov.br/saude/pt-br/assuntos/saude-de-a-a-z/a/arboviroses/informe-semanal/coe-dengue-informe-01-led_.pdf.^(6)^ Among the various interpretations on this significant increase in 2024, the response to climate change and its consequences is a priority.^(1,3,7)^

Some population groups are more susceptible to complications and progression to more severe forms of dengue, including pregnant and puerperal women,^(8)^ especially up to 14 days postpartum, as a result of the slow return of physiological adaptations to pre-pregnancy patterns. Preliminary data (not yet released) from SINAN/CGARB/MS indicate a considerable increase in the number of dengue cases in pregnant women in 2024. When comparing the recorded frequency of these diagnoses in epidemiological weeks 01-06 in year 2023 to the same weeks in year 2024, the number of dengue cases in pregnant women increased by 345.2%. Considering this increase in the number of pregnant women with dengue, combined with the risk of severe forms in these women, a worrying public health picture emerges, since both the maternal and perinatal prognosis can be compromised in this disease.^(9-12)^ Note there is still no progress regarding a specific treatment of dengue or safe vaccines for use during pregnancy.^(13,14)^

Objectively, accessible prophylaxis to avoid DENV infection is based on personal and community behavioral attitudes that must be continually remembered, reinforced and passed on to everyone, emphasizing that care against *Aedes aegypti* translates into expanded benefits, as it prevents all arboviruses transmitted by this mosquito.^(15-17)^

If prophylactic measures fail, care units for pregnant and puerperal women up to the 14^th^ day postpartum, still in the initial phase of the disease, must be properly organized to avoid progression to severe dengue, which can lead to death. In addition to user embracement, screening with risk classification is of paramount importance, enabling priority and timely treatment for all cases, especially those with warning or serious signs.^(18)^

All this information combined with the limitation of studies published specifically on clinical, antenatal, obstetric and puerperal care for women with dengue in our country justify the development of this Protocol, providing assertiveness and speed in the care of these women, potentially reducing maternal and perinatal health problems.^(10,19,20)^

## The dengue virus

Arboviruses are phylogenetically organized into six families (*Togaviridae, Flaviviridae, Bunyaviridae, Reoviridae, Rhabdoviridae,* and *Orthomyxoviridae*).^(21)^ Listing the arboviruses is crucial given their importance for the clinical and laboratory differential diagnosis of infection caused by DENV, one of the representatives of the *Flaviviridae* family.^(15,22-24)^ DENV is an RNA virus and has four different serotypes called DENV-1, DENV-2, DENV-3 and DENV-4, which can present diverse pathogenic potential and clinical manifestations of varied spectrum.^(25)^ The main vector of DENV in the urban cycle in Brazil is the *Aedes aegypti* mosquito, responsible for both epidemic outbreaks and endemic rates of this infection.^(23,26)^

## Influence of dengue on maternal and perinatal prognosis

All dengue serotypes increase the production of pro-inflammatory chemokines, promote thrombocytopenia and increase vascular permeability, pathophysiological phenomena responsible for several problems to the maternal health. Due to compromised vascular permeability, there is a greater risk of shock, thrombocytopenia and hemorrhagic phenomena aggravated by the increase in pro-inflammatory chemokines. The literature has shown that pregnant women are at greater risk of progressing to more severe forms of the disease and death.^(27)^

In Brazil, a retrospective evaluation showed that the rate of maternal deaths from dengue between 2007-2015 was four times higher among pregnant women with dengue, with a worse prognosis when occurring in the third trimester of pregnancy.^(9)^ Another evaluation of maternal mortality by dengue between 2007 and 2012 showed an increase in this rate by statistically significant values when comparing the first 10 days of the disease to deaths occurring after 10 days, indicating that women in the pregnancy-puerperal cycle need differentiated care and attention in the acute phase of the disease.^(10,28)^ When comparing maternal mortality by dengue in a prospective evaluation of 40 pregnant women in India, high rates of postpartum hemorrhage, acute kidney injury, liver failure, shock and maternal death in the puerperal period were found.^(29)^

Considering the rationale in which changes in the maternal organism are associated with possible ominous outcomes of dengue during pregnancy and how these changes remain more evident in the first two weeks after birth, the proposal of this Protocol is to also include puerperal women until the 14^th^ day postpartum in this higher risk group.

Physiological adaptations to pregnancy, such as hypotension and tachycardia may complicate the proper and early identification of clinical manifestations of dengue and delay diagnosis. The increase in capillary permeability in pregnant women is a functional adaptation, but it is clearly exacerbated in dengue. This fact warns of the greater risk of pregnant and puerperal women with dengue developing acute lung edema when included in a hyperhydration regime, which is part of the treatment of the disease. Particular attention is required with bleeding, making a differential diagnosis between those resulting from dengue and those linked to obstetric complications.^(30)^

The gestational and perinatal prognosis in pregnant women with dengue can also be compromised and bears a direct association with gestational age (worse in the third trimester), the intensity of the disease and its complications.^(9,31-33)^ The production of pro-inflammatory cytokines can stimulate the uterus, as in addition to increasing systemic capillary permeability and placental changes, it has the potential to cause abortion, prematurity, fetal growth restriction, fetal death and vertical transmission of DENV.^(11,34-37)^ Local structural changes in the placenta include edema of the villous stroma, syncytial nodes and chorangiosis, which can harm maternal and fetal exchanges occurring in these villi, in addition facilitating vertical transmission of the virus.^(38)^ High rates of miscarriage, prematurity, low birth weight, fetal and neonatal death were observed in a prospective evaluation of 40 Indian pregnant women with dengue.^(29)^

Although in general the vertical transmission of DENV is an infrequent event,^(39-41)^ when it occurs, prompt diagnosis is necessary, providing appropriate and rapid intervention for the newborn.^(20,42)^ According to Arragain et al. (2017),^(43)^ vertical transmission of DENV is more common when the period of maternal viremia is close to birth, and the postnatal evolution of these newborns presents high morbidity rates.

## Pathophysiology

There are still several gaps in the pathophysiological understanding of dengue.^(44)^ For several of the clinical manifestations of dengue, there are already very consistent pathophysiological mechanisms for understanding them.^(44)^ However, there are still several gaps in pathophysiological understanding of dengue.^(45)^ DENV infectivity depends on the binding of viral envelope glycoproteins to the preponderant cellular receptors on the surface of Langerhans cells,^(46)^ allowing the viral RNA to enter the cytoplasm and from hence, viral replication can occur. These cells are the preferential target of DENV, but also play an important role in taking the virus to the lymph nodes, where monocytes and macrophages are recruited, establishing the infection. From then on, the infection becomes systemic (underlying the entire pathophysiology of the disease) and the immune response is triggered.^(46)^

After an initial incubation period typically of 3-7 days, viremia increases and the infection manifests itself with a sudden onset of fever, known as the febrile phase.^(23)^ Some individuals pass to the critical phase, which is associated with accentuated plasma leakage, while others progress to the recovery phase without developing significant plasma leakage.^(47)^ The endothelial dysfunction observed in the critical phase of severe dengue is associated with a variable increase in vascular permeability, which is responsible for vascular leakage into interstitial space, causing accumulation of fluid in the pleural and peritoneal cavities, arterial hypotension and poor tissue perfusion.^(48)^ Many cytokine mediators,^(49,50)^ mast cell products, inflammatory lipid mediators, NS1 (which induces an increase in phospholipase A2) and platelet activating factor are some of the factors implicated in vascular leakage and the genesis of shock.^(51,52)^

The most frequent and important hematological changes in dengue are leukopenia, thrombocytopenia, and increased hematocrit, wich is a consequence of plasma leakage. Leukopenia occurs early in both mild and more severe forms of the disease and appears to be a result of a direct effect of the virus on the bone marrow. Some degree of thrombocytopenia is common in different clinical forms, but severe thrombocytopenia (<50,000 platelets/mm^3^) is associated with greater disease severity and unfavorable outcomes. Several mechanisms are suggested for the occurrence of thrombocytopenia in dengue. Among them are the reduction in thrombopoiesis secondary to bone marrow toxicity caused by DENV and the increase in the removal of platelets from the peripheral blood.^(53)^ The mechanisms underlying bone marrow suppression and the consequent thrombocytopenia may also be associated with functional changes in the cells of the stroma that regulate the secretion of inflammatory cytokines and hematopoiesis, quantitatively and qualitatively altering the production of platelets.^(54)^ It has also been demonstrated that the viral antigen NS1 induces activity of the enzyme phospholipase A2, prostaglandins and inflammatory cytokines in monocytes, contributing to thrombocytopenia.^(52)^ Other mechanisms suggested point to the adhesion of platelets to leukocytes, in addition to the production of antibodies.^(55)^ These changes are associated with the hemorrhagic events of dengue.

On average, 24 hours after DENV enters the body, the immune system begins to develop its response, initially based on the production of IgM, detectable for diagnostic support after the 5^th^ day of clinical manifestations of dengue.^(56)^ The production of IgG is normally detectable after 14 days, although the onset of IgG production can sometimes be anticipated. In cases of previous dengue infection, the immune response to the current infection is blocked, as the organism searches cellular memory for a new response to DENV from the old infection. This is one of the explanations for the increasing severity in cases of recurrent dengue, predisposing to severe dengue.^(57)^ In cases of recurrent dengue, the available information indicates that the longer the interval between infections, the greater the severity.^(58)^

Although DENV infection is a self-limited disease in most individuals, presenting with mild symptoms,^(19)^ it is estimated that 3-5% of cases will progress to more severe forms and severe dengue may affect 1% of people with the infection. As evolution is unpredictable, initial good quality care must always be provided for all pregnant women. Although there will be asymptomatic cases of dengue, when symptomatic, the disease is characterized as systemic and dynamic with a wide clinical spectrum, ranging from oligosymptomatic forms to serious conditions such as hemorrhages, shock and significant failure of other organs, potentially lethal.^(44)^ Three clinical phases are recognized in its symptomatic evolution; febrile, critical and recovery. Knowing them is essential for the appropriate management of the different clinical forms, especially among pregnant and puerperal women up to 14 days postpartum.^(26)^

## Diagnosis

The procedures used to diagnose infections caused by DENV in pregnant and puerperal women do not differ from those used in the adult population outside the pregnancy-puerperal cycle. The classic division is into clinical diagnosis (epidemiology, information from anamnesis and clinical examination), differential diagnoses, diagnosis of the severity of the case and laboratory diagnosis. As a basic premise of care for pregnant and puerperal women up to 14 days postpartum, given the similarity between the clinical manifestations of some infections (other arboviruses, Covid-19, leptospirosis and measles, among others) and the possibility of rapid evolution of infections by the various types of DENV to severe forms very quickly, in endemic areas, the infection resulting from DENV should be considered as the main differential diagnosis between them, allowing the quick and assertive clinical support measures.

## Clinical diagnosis

For the clinical diagnosis of dengue in pregnant women, it is important to highlight that some of the signs and symptoms of the disease may be confused with common changes in pregnant women such as nausea, vomiting, abdominal pain, postural hypotension and tachycardia, delaying diagnosis and early hydration measures. As seen, the clinical diagnosis of dengue is extremely important, signaling the immediate start of the therapy in the event of a suspected clinical condition.^(30)^ Classic tools are used (anamnesis and clinical examination) in the clinical diagnosis of dengue, with adaptations that the disease demands, like epidemiological variables. Given the broad spectrum of signs and symptoms, clinical diagnosis of dengue infection can be challenging. Its clinical picture can vary from a simple low-intensity febrile manifestation to serious and potentially fatal syndromes.^(59)^ Therefore, when faced with a suspected clinical picture of dengue in a pregnant woman, laboratory diagnosis is essential, but should not delay the early and effective therapeutic management, variables that contribute to reduce morbidity and mortality resulting from dengue.^(23,57,60)^

## Epidemiological variables

Emphasizing epidemiological aspects as an important part of the diagnosis, we recall the high relevance of DENV infection for humans, which is a growing challenge in tropical and subtropical regions of the planet. However, globalization has made this disease migration to temperate climate regions in recent years, precisely as a result of the increase in arthropod vectors in these latitudes.^(3,61)^ Through anamnesis, the health professional must be aware of symptomatic pregnant and puerperal women returning from these areas (less than 14 days).^(62)^ Likewise, consider the possible coincidence of epidemiological scenarios where the frequency of measles, Covid-19 and leptospirosis cannot be ruled out.^(21,63)^

As the basic risk of the occurrence of arboviruses is the presence of the vector (*Aedes aegypti*), the appearance and circulation of a given arbovirus initially depends on the introduction of this virus into that community and from then on, on the existing sanitary conditions. According to epidemiological data released by the Ministry of Health,^(5,26)^ the Brazilian population may be considered at constant risk of dengue.

### Anamnesis and clinical examination

In addition to offering epidemiological information about dengue, through the anamnesis, it is possible to characterize the presence of manifestations and define which phase of the disease the pregnant woman is in. It also allows access to the first information regarding the different possible diagnoses. In the history of the current illness related to dengue, the clinical manifestations depend on the phase of the disease.^(18,59,60)^ In the initial phase, clinical manifestations are dominated by fever, headache, maculopapular rash, malaise, asthenia, myalgia, arthralgia (usually of low intensity), retro-orbital pain, nausea, vomiting and diarrhea. Although in more serious cases the fever tends to subside between the 3^rd^ and 7^th^ day (defervescence period), other clinical manifestations appear, which should alert the obstetrician to a more serious development. These are cases of intense abdominal pain, persistent vomiting, effusions of virtual cavities, hypotension, hepatomegaly, hemorrhagic manifestations and signs of central nervous system impairment (lethargy and/or irritability). Details of these manifestations will be discussed in the section on assessing the severity of dengue.

Considering the physical examination, in most cases, the information from anamnesis is confirmed and the findings will depend on the stage of the disease. The following should be systematically assessed: temperature, heart rate, pulse characteristics (rate, full or filiform), level of consciousness (Glasgow coma scale), hydration, blood pressure, capillary refill time, virtual cavity effusions, characterization of abdominal pain, hepatomegaly, the presence of a rash, and spontaneous or induced hemorrhagic manifestations (tourniquet test with more than 20 petechiae in the area of the forearm defined for the test).^(18)^

Along with the clinical information, two indirect subsidiary tests complement the initial clinical suspicion of dengue, especially in pregnant women, the tourniquet test and the blood count. Despite its limitations in severe cases and in obese patients, the tourniquet test is indirect evidence of capillary fragility, the basis of dehydration to varying degrees, of the reduction in the formed elements of the blood and high hematocrit. The blood count provides important information such as the indirect aggression of the marrow by viral action, with a reduction of elements of the blood (notably thrombocytopenia) and the high hematocrit resulting from the fluid leakage that occurs in response to the increase in capillary permeability. According to guidance from the Ministry of Health,^(18)^ every suspected case of dengue must be reported at http://sinan.saude.gov.br/sinan/login/login.jsf

### Differential diagnosis

Some clinical manifestations of typical complications of the pregnancy-puerperal cycle can make it difficult to evaluate pregnant and puerperal women infected with DENV, such as hyperemesis gravidarum, preeclampsia (especially more severe conditions), HELLP syndrome, chorioamnionitis, and puerperal infection.^(19)^

As the clinical manifestations of dengue are similar to those of a large number of diseases, its differential diagnosis has a wide range of possibilities. Expanded information on all possibilities, providing alerts from this regional perspective is the most appropriate approach. Given these considerations, the list may include other arboviruses (zika, chikungunya), influenza, Covid-19, leptospirosis, viral hepatitis, malaria, rubella, measles, scarlet fever, infectious erythema, infectious mononucleosis, hantavirus, yellow fever, rickettsioses (fever maculosa), parvovirus, cytomegalovirus, enteroviruses, septic processes, encephalitis/meningitis (viral and bacterial) and typhoid fever. Purpura, hematological neoplasms, food poisoning and pharmacodermia also cause some diagnostic difficulties.^(18,63)^

Considering the diseases that cause abdominal pain, some diagnoses need to be ruled out as appendicitis, intestinal obstruction, liver abscess, acute abdomen, pneumonia, urinary tract infection, and acute cholecystitis, among other conditions. Myocarditis (of various causes) also appears on this list, generally linked to severe dengue conditions, including shock.

Given the several clinical characteristics in common, at the epidemiological moment in Brazil, the infections that most challenge differential diagnosis are other arboviruses (zika and chikungunya),^(5-7)^ Covid-19, leptospirosis, influenza and measles,^(63)^ resulting in a demand for laboratory confirmation in most cases. The clinical manifestations of the arboviruses discussed here differ more by the intensity of clinical manifestations than by their presence.^(24,64)^

In places where it is difficult to access laboratory tests to confirm the diagnosis of dengue in pregnant women, it can be inferred with a high degree of accuracy based on anamnesis, physical examination, tourniquet test and differential diagnosis, allowing a quick start of the first therapeutic support measures.^(18)^ A more accurate assessment of the severity of the case will be possible by carrying out a blood count, while in places with more laboratory resources, other tests will be indicated, including the etiological diagnosis of the infection (NS1).

#### Laboratory diagnosis

Two objectives are considered when talking about laboratory resources to support the care of pregnant and puerperal women up to the 14^th^ day postpartum with dengue. The first is the etiological diagnosis of the infection and the second refers to laboratory resources to assess the severity of the disease.

#### Etiological laboratory diagnosis

The most suitable tests for the etiological laboratory diagnosis of the initial phase of dengue are those that identify specific viral particles, such as non-structural glycoprotein 1 (NS1) or viral RNA in the blood.^(65)^ In the acute phase, which occurs in the first 3-7 days after the onset of symptoms, detection of serum viral RNA would be the best test by using reverse transcription polymerase chain reaction (RT-PCR) as the most accurate test for diagnosing dengue,^(66)^ although its accessibility is still limited for patients to obtain a quick diagnostic response from the Brazilian Universal Public Health System (SUS) patients. In several parts of the country, these samples still need to be sent to centralized official laboratories, which delays the diagnostic process.^(62)^ Some assays can diagnose dengue, zika and chikungunya in a single sample. The suggestion is, before obtaining the sample, checking the forwarding flow to the laboratory in which it will be processed.

By its performance, practicality and quick results, testing for the NS1 antigen in maternal blood is the most used technique for the rapid laboratory diagnosis of dengue.^(65)^ The NS1 antigen is a highly conserved glycoprotein that appears to be essential for the viability of the virus and appears in both primary and secondary infections. However, the NS1 detection rate is higher in sera from patients with primary infection (75%-97%) compared to sera from individuals with secondary infection (60%-70%). Various techniques can be used to detect this antigen, including immunochromatography or ELISA. From a practical assistance point of view, the difference between these types of exams is the time interval until the exam results are released. The immunochromatographic technique is used for rapid diagnostic testing, as its result is possible in less than two hours. The immunoenzymatic technique is more sensitive, but the results take longer.^(67,68)^ NS1 testing presents excellent results when carried out between the 3^rd^ and 5^th^ day of the disease, and in some cases, it may present positive results earlier. The NS1 normally becomes negative after the 7^th^ day, when the production of antibodies neutralizes this antigen.^(22)^

From the 6^th^ day of clinical manifestations of dengue, the laboratory diagnosis of the infection is made based on tests of the immune response to the virus, measuring the presence of IgM and IgG. Immunoenzymatic assays are the most used for the serological diagnosis of DENV infection in pregnant women, as they can measure IgM (from the 7^th^ day) and IgG (from 14-17 days). These tests may be positive earlier in some cases. The concentration of these antibodies slowly increases after this period and supposedly persists for life. In patients with secondary infections, anti-dengue IgG titers increase rapidly within the first week of illness. In cases of clinical manifestations compatible with DENV infection, but with negative NS1 and RT-PCR results, or if more than 7 days have passed since the onset of symptoms, a serological test is indicated to identify immunoglobulin M (IgM) for DENV and for ZIKV. Note there are some difficulties in its interpretation, mainly those arising from the cross-reaction to infections caused by other *Flaviviruses*, mainly ZIKV.^(62,69)^

## Laboratory diagnosis to assess the severity of dengue

The tests used to assess the degree of systemic involvement of pregnant women with dengue in the febrile phase begin with the blood count, highlighting hematocrit, leukopenia and thrombocytopenia.^(22)^ High hematocrit is directly related to the degree of vascular leakage, while the severity of thrombocytopenia is closely associated with hemorrhagic conditions. Remember that the hemodilution typical of pregnancy may mask the thrombocytopenia, leukopenia and hemoconcentration associated with dengue, and these changes regress slowly after birth.

If the case progresses to the critical phase of dengue, laboratory tests will support the clinical diagnosis of the affected organs. In general, liver function (ALT/ASL transaminases, bilirubin), metabolic function (glycemia, arterial gases), renal function (sodium, potassium, albuminuria, creatinine and urea) and cardiac function (enzymes and electrocardiogram) should be evaluated. Imaging tests complete the laboratory tests and must be requested according to clinical demand. If neurological/cognitive function is altered, magnetic resonance imaging or tomography are recommended. A simple radiological examination is indicated to assess impairment of lung function. Pericardial and abdominal effusions are best evaluated by ultrasound. There is no restriction on carrying out these exams in pregnant women, including radiological exams with abdominal protection.

Laboratory resources are an essential support in the differential diagnosis of severe malaria, leptospirosis, fulminant viral hepatitis, other flavivirus infections, HELLP syndrome and exogenous intoxication.^(16,21,60)^

Ultrasound examination and cardiotocography are used to assess fetal well-being depending on gestational age and the degree of fetal health impairment. More details in the specific section of this Protocol.

## Clinical phases of dengue and characterization of severity

**Figure 2:**
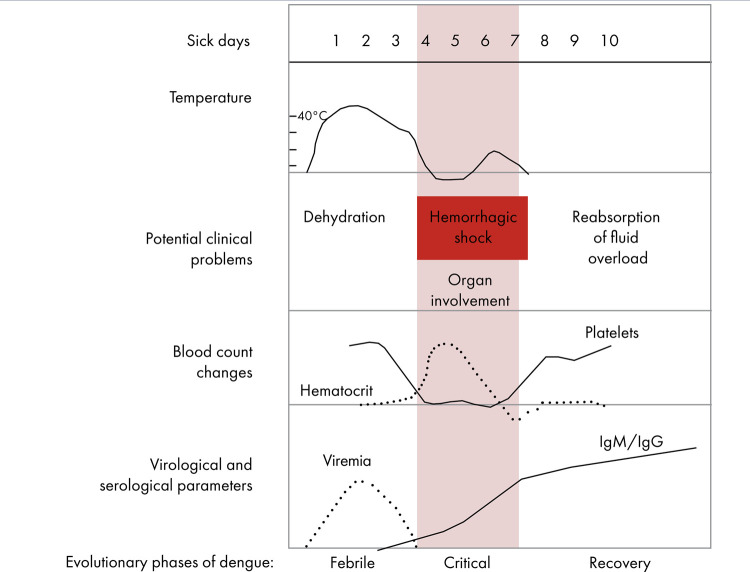
Evolutionary phases of dengue according to evolution time

Source: Adapted from the World Health Organization (2009).^(60)^

The pathophysiological changes of dengue are dynamic and translate the disease into its clinical phases or stages. The first phase is called febrile, the second is the critical phase and the third is recovery phase (Figure 2).^(18,23,60)^

## Febrile phase

This phase, also called “dengue without warning signs”, is characterized by the presence of fever, generally of sudden onset and high (39-40ºC), associated with headache, maculopapular skin rash (50% of cases), prostration, myalgia, low-intensity arthralgia and retro-ocular pain. Anorexia, nausea, vomiting and diarrhea with loose stools may also be present. In general, the tourniquet test is positive at this stage. Most patients recover after the febrile phase with an improvement in fever and a gradual return to normality.^(59)^ Although these pregnant women do not require hospitalization, they need to be cared for in health care environments where hydration can be controlled and blood counts requested.

## Critical phase

The critical phase of dengue is subdivided into “Dengue with warning signs” and “Severe dengue”. Evolutionarily, this phase begins with the onset of warning signs and if the disease is not controlled, clinical and laboratory manifestations of severe dengue appear. Outside and during the pregnancy-puerperal cycle, warning signs must be systematically tested and valued, as they indicate changes with the potential for progression to more severe forms of the disease and worsening of maternal health. Normally, the critical phase involves progressive leukopenia and thrombocytopenia.^(18)^ These cases require greater care and a hospital setting with admission is indicated.

## Dengue with warning signs

In the presence of warning signs, rapid and accurate medical care becomes essential. In general, warning signs begin with the decline of fever at 3-7 days after the onset of symptoms. Warning signs result from increased vascular permeability and mark the beginning of clinical deterioration, a condition that will require hospitalization. Chart 1 shows the warning signs of dengue.^(18, 60)^

**Chart 1:** Dengue warning signs


Severe and continuous abdominal pain Persistent vomiting Accumulation of liquids in virtual cavities Postural hypotension and/or lipothymia Hepatomegaly (liver > 2cm below the costal margin) Mucosal bleeding Signs of Central Nervous System involvement (lethargy, irritability, behavioral changes, seizures, among others) Increase in hematocrit (>10%) and drop in platelets

Source: Adapted from the World Health Organization (2009).^(60)^

The degree of vascular permeability varies. At this point, patients with lower permeability show clinical improvement, while those with greater permeability can progress to more severe forms.^(21)^

## Severe dengue

This stage is characterized by intense plasma leakage, predisposing clinical conditions such as ascites and pleural effusion that can be noticed clinically. It progresses with increased hematocrit (higher hematocrit, greater severity) and hypoalbuminemia. Effusions from virtual cavities are easily confirmed by imaging tests. Below, other serious forms of dengue will be referred to as shock, hemorrhages and involvement of other organs (Chart 2).^(18, 60)^ These cases require advanced care in an intensive care environment.

**Chart 2:** Signs of dengue severity


Severe plasma leakage, leading to shock evidenced by tachycardia Cold distal extremities with weak and filiform pulse; delayed capillary refill (> 2 seconds) Differential pressure <20mmHg (convergent BP) Arterial hypotension and cyanosis (late phase of shock) Tachypnea Hypothermia or sudden reduction in body temperature Oliguria (< 1.5 ml/kg/h) Acute edema with respiratory failure Severe bleeding that may result in hematemesis and/or melena Severe involvement of multiple organs

Source: Adapted from the World Health Organization (2009).^(60)^

In dengue, the shock is quick to set in and short-lived. It is usually preceded by warning signs, which may be due to both bleeding and plasma leakage and occurs between the 4^th^ and 5^th^ day of infection. It can lead to the patient’s death within 12 to 24 hours, requiring support and appropriate and immediate therapy due to the risk of disseminated intravascular coagulation, metabolic acidosis, cardiac and pulmonary dysfunction.

The hemorrhagic condition can occur without prolonged shock, and is also one of the severity criteria. In turn, organ involvement includes myocarditis, liver failure, respiratory failure, renal failure (rare, but with a worse prognosis) and involvement of the central nervous system (behavioral manifestations, seizure, and loss of consciousness, as an expression of viral encephalitis).^(60)^

Severe dengue occurs in less than 1% of people with dengue. Some risk factors are associated with severity, such as dengue caused by DENV-2 and previous infection, probably reflecting the secondary immune response, with acceleration in the formation of immune complexes and cellular immune response. In addition, the longer the interval between infections the greater the risk of severe dengue.^(70)^

## Recovery phase

In patients who have passed through the critical phase with or without progression to the severe form, there will be gradual reabsorption of the leaked fluids with progressive clinical improvement and increased urine output. The recovery phase is known to last 2-4 days, but fatigue may remain for a few weeks.

## Clinical management

Pregnant women with dengue should be treated according to the clinical phase of the disease and their particularities. Systematizing, grouping and stratifying certain organic changes that occur in pregnant women with dengue makes it easier to approach and develop clinical care protocols that can be followed in the most remote communities in the country. The first stratification is based on clinical manifestations and their severity, allowing that pregnant women with the same clinical manifestations and possible complications are grouped (Groups A, B, C and D).

### Formation of groups according to the severity of the disease

According to guidance from the Ministry of Health (2024),^(18)^ adapting guidelines from the WHO (2009)^(60)^ and PAHO (2016),^(23)^ patients are grouped according to the severity of the disease for greater assertiveness in clinical management as the follows:

**•**Group A: Meets the dengue requirements, but does not present any warning signs, without special clinical conditions, social vulnerability or comorbidities.

**•**Group B: Dengue without warning signs, but presents some special condition, social vulnerability or comorbidities. If there is spontaneous skin bleeding or bleeding induced by the tourniquet test, it is also classified in this group.^(18)^ Special clinical conditions and/or social risk or comorbidities are: pregnant women, infants (< 2 years), adults over 65 years of age, high blood pressure or other serious cardiovascular diseases, diabetes mellitus, chronic obstructive pulmonary disease, obesity, chronic hematological diseases (sickle cell anemia and purpura), chronic kidney disease, liver or autoimmune diseases.

**•**Group C: When there is any warning sign (Chart 1).

**•**Group D: When there is any sign of severity (Chart 2)

### Risk classification for the care of pregnant and puerperal women with dengue

The risk classification of pregnant and puerperal women suspected or diagnosed with dengue is essential to prioritize care based on the severity of the clinical condition. For these women, it is crucial to speed up care by reducing the waiting time for assistance. Data from anamnesis and physical examination are used in this stratification and guide the most appropriate therapeutic measures, considering the presence of warning signs and severity (Chart 1, 2 and 3).

**Chart 3:** Risk classification according to signs and symptoms

Blue	Group A	Service according to arrival time
Green	Group B	Priority (Pregnant and puerperal women up to the 14^th^ day postpartum)
Yellow	Group C	Urgency Need to be cared as quickly as possible
Red	Group D	Emergency Need for immediate care

Source: Adapted from Brazil (2024).^(18)^

### Clinical management according to severity classification for pregnant and puerperal women with dengue

Dengue severity criteria are identified through anamnesis, physical examination, blood count, and by the early recognition of warning and severity signs. These define the staging of the disease (Groups A, B, C and D) that will guide therapeutic interventions for pregnant women in each specific group. Once the severity criteria have been established, therapeutic interventions for pregnant and puerperal women up to the 14^th^ day postpartum must be promptly instituted according to the clinical staging of the disease. Both pregnant and puerperal women up to the 14^th^ day postpartum require constant surveillance, regardless of the severity, but at a specific pace to each phase. Even though tests to confirm dengue are mandatory for pregnant women, they are not essential for starting treatment at any stage of the disease.

In general terms, as there is no antiviral treatment for dengue, it will mostly consist of rest, ingestion or infusion of fluids to prevent dehydration, use of analgesics and antipyretics such as paracetamol or dipyrone, when necessary. The use of ASA (acetylsalicylic acid) and non-steroidal anti-inflammatory drugs (NSAIDs) are contraindicated, as they can worsen the bleeding condition. Always be prepared for the possibility of disease progression.^(30)^

Chart 4 summarizes the guidelines for oral hydration for people with dengue, which is indicated for patients in groups A and B. Note that pregnant and puerperal women up to the 14^th^ day postpartum are part of Group B.^(18)^

**Chart 4:** Guidelines for oral hydration of pregnant and puerperal women up to the 14th day postpartum with dengue


Oral hydration for pregnant and puerperal women should be started in a healthcare environment as soon as possible. Intake of 60 ml/kg/day; 1/3 with oral rehydration salts (ORS) and a larger volume at the beginning. For the remaining 2/3, advise the intake of homemade liquids (water, fruit juice, homemade saline solution, teas, coconut water, among others), using the most appropriate means to the patient’s eating habits. Specify the volume intake per day. For example, the recommendation for a pregnant or puerperal woman weighing 70 kg is the intake of 60 ml/kg/day, totaling 4.2 liters/day. Thus, a volume of 1.4 liters will be ingested in the first 4 hours. The remaining 2.8 liters will be distributed over other periods. In the first 4 hours of service, consider offering 1/3 of the volume. For pregnant and puerperal women who cannot tolerate oral hydration, infuse 2 to 4 ml/kg/hour of saline solution, following the principles of venous hydration control. Intolerance or refusal of oral hydration can even be a reason for hospitalization. Food should not be interrupted during hydration, but allowed according to the acceptance of the pregnant or puerperal woman.

Source: Adapted from the Ministry of Health (2024).^(18)^

**Figure 3:**
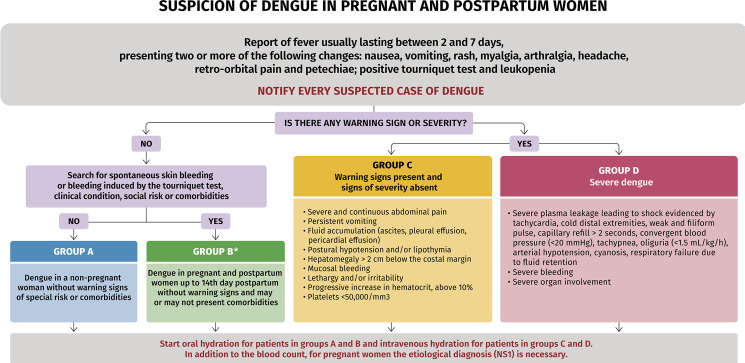
Flowchart of group formation according to stratification of dengue severity in pregnant and puerperal women up to the 14th day postpartum

Source: Adapted from the Ministry of Health (2024).^(18)^

**Figure 4:**
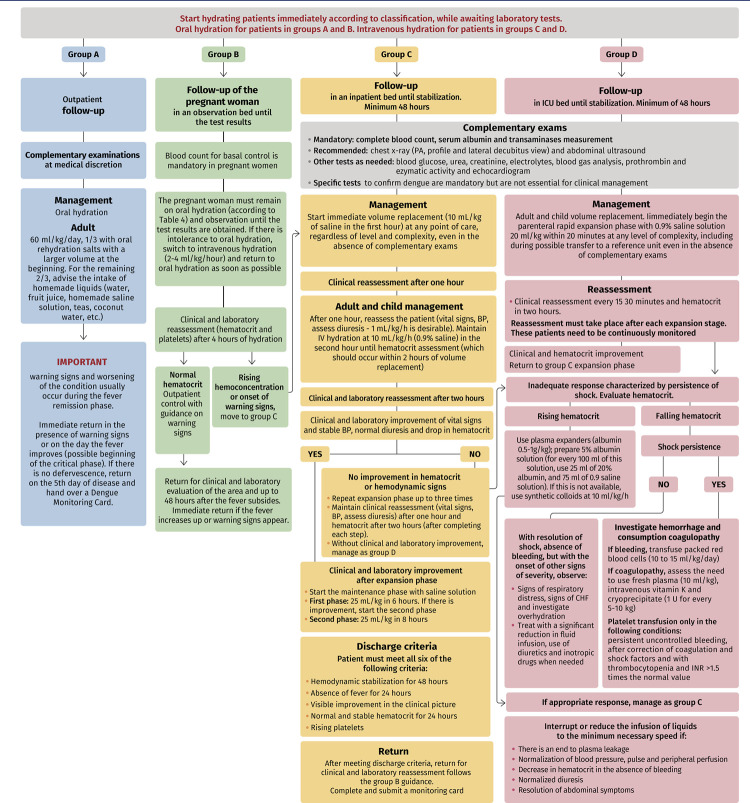
Flowchart for the evolutionary control of clinical care for pregnant and puerperal women (up to the 14th day postpartum) with dengue

Source: Adapted from the Ministry of Health (2024).^(18)^

Figures 3 and 4 show the flowcharts published by the Ministry of Health (2004)^(18)^ for the care of patients with dengue adopted in this Protocol, with the necessary adaptations for the care of pregnant and puerperal women up to the 14^th^ day postpartum.

Hospitalization of pregnant and puerperal women until the 14^th^ day postpartum will also be necessary if outpatient follow-up is impossible, in the presence of adverse social conditions or the concomitant occurrence of decompensated or difficult-to-control comorbidities such as diabetes mellitus, severe heart disease, high blood pressure, heart failure , use of anticoagulants or antiplatelet agents, asthmatic crisis and sickle cell anemia.^(18)^

### Group A. Non-pregnant, no warning signs

The management of non-pregnant women follows the recommendations of the Ministry of Health’s Manual on Dengue in Adults and Children.^(18)^ Pregnant women are among special clinical conditions and their inherent risk places them directly in Group B.

### Group B – Pregnant and puerperal women up to the 14th day postpartum without warning signs

Primarily, pregnant or puerperal women who present suggestive clinical manifestations or a diagnosis of dengue and do not present any signs of alarm or severity are included in Group B. They must be treated as quickly as possible and require surveillance and hydration. The aims of this measure are the faster maternal recovery and avoiding the risk of progression to more severe forms that harm the maternal and perinatal prognosis. In places where the mosquito vector is present, this pregnant/puerperal woman is considered a source of infection during the feverish period. For this reason, all suspected cases of dengue or other arboviruses must be kept in accommodation with protected windows and doors at home or in hospitals.^(62)^ The initial management of pregnant women in Group B is detailed below (Chart 5).

**Chart 5:** Care of pregnant and puerperal women up to the 14th day postpartum with dengue (Group B)


Request additional tests according to the associated clinical condition. Request a blood count to assess the platelet count and compare the hematocrit with the baseline value (the pregnant woman must have this information noted in her antenatal record card). If the hematocrit is up to 10% higher than its baseline: repeat the blood count daily within 48 hours after the fever stops. In the absence of baseline hematocrit, consider a hematocrit of 38-40%. Keep the pregnant woman in an observation bed (strict control of vital signs, signs and symptoms) until the test results are checked; Start oral hydration: intake of 60 ml/kg/day, 1/3 in the first 4 hours (details in Chart 4). Repeat clinical, hematocrit and platelets assessment after 4 hours of hydration. Symptomatic medications: antipyretics, analgesics and antiemetics, as needed. Non-steroidal anti-inflammatory drugs are contraindicated. Patients with normal hematocrit can undergo outpatient control with daily clinical and laboratory reassessment (up to 48 hours after the fever has stopped); advise on warning signs, bleeding or shock and advise not to self-medicate; remain at rest and seek emergency services in case of bleeding or warning signs. In the presence of hemoconcentration (increase in hematocrit > 10% or value > 44% when there is no previous test for comparison) or the onset of warning signs, classification into Group C is automatic and the pregnant or puerperal woman up to the 14^th^ day postpartum must be hospitalized. Platelet count < 50,000/mm^3^ is also an indicator of severity, and hospital admission is recommended for better control.

Source: Adapted from the Ministry of Health (2024).^(18)^

### Group C – Pregnant and puerperal women up to the 14th day postpartum with dengue and warning signs

Pregnant women who present any warning signs (see Chart 1) are classified in Group C. They should preferably be admitted to a hospital where there is the possibility of intensive care, in case the disease progresses. The management of pregnant women in Group C is summarized in chart 6.

**Chart 6:** Care of pregnant and puerperal women up to the 14th day postpartum with dengue and warning signs (Group C)


They must remain hospitalized until stabilization, at least 48 hours. Start immediate intravenous volume replacement (10 ml/kg of saline in the first hour) at any point of care, regardless of the level of complexity, including during eventual transfer to a reference unit, even in the absence of complementary exams. Monitor vital signs during liquid infusion, especially heart and respiratory rate, respiratory auscultation, pulse quality, jugular turgidity, diuresis and level of consciousness. Request blood count, electrolytes, serum albumin measurement, transaminases. Request chest X-rays (with adequate abdominal protection) and abdominal ultrasound. Depending on the need, request blood glucose, urea, creatinine, blood gas analysis, activated partial thromboplastin time and echocardiogram. Reevaluate after 1 hour (vital signs, blood pressure, diuresis - desirable 1ml/kg/h), maintain hydration 10 ml/kg/h in the second hour until hematocrit assessment, which should occur in 2 hours (after stage of volume replacement). The maximum total for each expansion phase is 20 ml/kg in two hours with gradual and monitored administration. If there is no improvement in hematocrit or hemodynamic signs, repeat the expansion phase up to 3 times. Clinical reassessment (vital signs, blood pressure, diuresis) after 1 hour and hematocrit reassessment in 2 hours after completing each step. If there is clinical and laboratory improvement after the expansion phase, start the maintenance phase: First phase: 25 ml/kg in 6 hours, if there is clinical improvement, start the second phase. Second phase: 25 ml/kg in 8 hours, 1/3 with saline solution and 2/3 with glucose solution. Pregnant women in Group C must remain hospitalized until stabilization and discharge criteria for a minimum period of 48 hours. If there is no clinical and laboratory improvement, manage as Group D.

Source: Adapted from the Ministry of Health (2024).^(18)^

### Group D – Pregnant and puerperal women up to the 14th day postpartum with dengue and signs of severity

Pregnant and puerperal women who present signs of severity (shock, severe bleeding or severe organ dysfunction) will be classified in Group D. Signs of shock in dengue are: tachycardia, cold distal extremities, weak filiform pulse, delayed capillary refill time (>2 seconds ), convergent blood pressure (<20 mmHg), tachypnea, oliguria (<1.5 mL/kg/h), arterial hypotension (late shock phase) and cyanosis (late shock phase). Chart 7 summarizes the care of pregnant women in Group D.

**Chart 7:** Care of pregnant and puerperal women up to the 14th day postpartum with dengue and signs of severity (Group D)


Request an intensive care bed. If it is not accessible, immediately institute management and monitoring measures. For volume replacement, immediately begin the rapid parenteral expansion phase with 0.9% saline solution (20 ml/kg in up to 20 minutes) at any level of complexity, including during possible transfer to a reference unit, even in the absence of additional examinations. Clinical reassessment every 15-30 minutes and hematocrit assessment every 2 hours. These patients require continuous monitoring. Repeat expansion phase up to three times. If there is clinical and laboratory improvement after the expansion phase, return to the Group C expansion phase and follow the recommended management. Request a complete blood count and albumin and transaminase levels. Request chest X-rays (with adequate abdominal protection) and ultrasound to evaluate cavitary effusions. Other tests such as blood glucose, urea, creatinine, electrolytes, blood gas analysis, prothrombin time and enzyme activity and echocardiogram may be performed as necessary. These patients must remain monitored in an ICU bed until stabilization (minimum of 48 hours) and after stabilization, they must remain in a hospital bed If the response is inappropriate and characterized by shock, it will be necessary to assess: If rising hematocrit after appropriate volume replacement, use plasma expanders (albumin 0.5 g/kg to 1 g/kg); prepare 5% albumin solution (for every 100 ml of this solution, use 25 ml of 20% albumin and 75 ml of 0.9% saline). If not available, use synthetic colloids (10 ml/kg/hour); If falling hematocrit and persistence of shock, investigate occult hemorrhages and evaluate coagulation; In the presence of hemorrhage: transfuse packed red blood cells (10 to 15 mL/kg/day); In the presence of coagulopathy: assess the need to use fresh plasma (10 mL/kg), intravenous vitamin K and cryoprecipitate (1U for every 5 kg to 10 kg); Consider platelet transfusion in the following conditions: persistent uncontrolled bleeding, after correction of coagulation factors and shock; thrombocytopenia and INR >1.5 times the normal value. If the hematocrit is falling with resolution of the shock, absence of bleeding, but with the onset of other signs of severity, observe: Signs of respiratory distress, signs of congestive heart failure and investigate overhydration; Reduce fluid infusion, use of diuretics and inotropic drugs, when necessary. The infusion of liquids must be interrupted or reduced to the minimum necessary speed if: There is an end to plasma leakage; Normalization of blood pressure, pulse and peripheral perfusion; Decrease in hematocrit in the absence of bleeding; Normalized diuresis; Resolution of abdominal symptoms. After meeting the discharge criteria, the return for clinical and laboratory reassessment follows group B guidance.

Source: Adapted from the Ministry of Health (2024).^(18)^

According to the criteria of the Ministry of Health,^(18)^ hospital discharge for pregnant and puerperal women with dengue follows the same discharge guidelines as those for other patients, which include: absence of fever, elevated platelets, improvement in general condition, normal hematocrit, stable in the prior 24 hours and hemodynamic stabilization in the last 48 hours. In addition, it is also necessary to evaluate the safety and social support for this pregnant or puerperal woman.

## Antenatal, obstetric and puerperal care line

Since the use of the available vaccine against dengue is restricted in pregnant and lactating women because it contains live, attenuated viruses, it is imperative to seek alternatives to prevent this infection during antenatal care. Regardless of the options to be adopted, these recommendations are mandatory during antenatal consultations.

### Preventive care for dengue in antenatal care

Regular antenatal care does not differ if a given community or region is experiencing a peak dengue epidemic period. The only differences are related to the emphasis given to disease prevention guidelines and the provision of information that allows pregnant women to promptly identify the signs and symptoms of dengue and seek medical assistance when they are present. This information should always address care in controlling the breeding sites of mosquitoes of the genus *Aedes*, even though we know its results are limited. Preventing mosquitoes from entering homes are also recommendations of limited scope, as this is a high-cost initiative (screens on doors and windows), but must also be mentioned. Faced with this reality of failures and limitations, only behavioral measures remain feasible and with some result in practice, such as the use of light clothing (covering as much of the exposed body surface as possible) and the use of repellents. Actually, the measure with the greatest impact on reducing dengue cases is the use of repellents (including Icaridin, DEET and IR3535). Details of these guidelines are part of the Prevention section of this Protocol.

### Clinical and obstetric care in the acute phase of dengue

One of the obstetrician’s main precautions when caring for pregnant and puerperal women (up to the 14^th^ day postpartum) with dengue is surveillance so that clinical management complies with the criteria outlined in the Clinical management section of this Protocol. Note that hydration for pregnant women is the most effective therapeutic strategy in preventing and controlling the most serious clinical forms. Hydration is the shortest way to correct the effects of interstitial fluid leakage, potentially also helping to correct thrombocytopenia.

In cases of pregnant or puerperal women with dengue who require prolonged hospitalization, walking should be encouraged as a strategy to prevent venous thromboembolism, which already has increased occurrence during pregnancy and tends to worsen in cases of altered coagulability present in dengue. Although hemorrhagic phenomena are the most common in dengue, cases of thrombosis can arise from hemoconcentration and blood hyperviscosity. Consider physiotherapeutic measures, lower limb massage and compressive or pneumatic stockings to avoid them if the pregnant woman is confined to bed.

In general, after the start of clinical procedures, the obstetrician’s role is more of expectant management in these cases, restricted to the assessment of maternal well-being (seeking to comply with all clinical care guidelines) and fetal well-being. In the opinion of Chong et al. (2023),^(12)^ all complications and situations that occur in pregnant women with dengue, such as preeclampsia, premature placental abruption, use of antiplatelet or anticoagulant drugs and pre-term labor need to be better studied. The current protocols assume common sense in most cases, aiming at the most logical conduct, most of the time still without the support of specific research.^(57)^

Some situations require attention because they are not addressed in the care protocols for pregnant and puerperal women with dengue reported in the literature. The first of these is intolerance or resistance to oral hydration recommended for the treatment of pregnant women in Group B. As balanced hydration of the pregnant woman is one of the success factors of the treatment, intravenous hydration is then chosen (2-4 ml/Kg/hour), maintaining all control precautions to avoid overhydration and acute pulmonary edema, seeking to restore the oral route as soon as possible.^(12)^ As the risk of acute pulmonary edema during hydration is a possibility in this scenario, it is important to record the monitoring of the pregnant woman’s vital signs during fluid infusion, especially heart and respiratory rates, respiratory auscultation, pulse characteristics, jugular turgidity, diuresis and level of consciousness.

Another variable of concern in the care of pregnant women with dengue is the use of ASA for secondary prevention of preeclampsia or to avoid thromboembolic phenomena. The use of this medication should be suspended during the acute phase of dengue. Although there are no studies on the relevance of returning this medication throughout the pregnancy sequence, it is common sense that ASA can be reintroduced one week after disease remission with the expectation that the protective benefits have been maintained during the interruption of prophylaxis and will be restored upon its return. The use of antiplatelet agents (ASA or clopidogrel) as part of the therapy of diseases with a high thromboembolic risk (such as connective tissue diseases and acquired thrombophilia) is more complicated. In these cases, the tendency is also to suspend the medication in the acute phase of dengue, but the pregnant woman must be hospitalized for daily control.

The situation of pregnant women with dengue who use anticoagulants prophylactically also brings some uncertainty to the obstetrician. The best approach is to individualize the case, evaluating the indication of the anticoagulant, the risk of thromboembolism (for example, pregnant women using metal valve prostheses) and the severity of dengue (risk of hemorrhage). Discontinuation of the anticoagulant is always indicated when there is bleeding or the platelet count is less than 30,000/mm^3^. The INR assessment must occur on a daily basis in these cases, and the medication should be resumed when the infectious process has resolved.

A relatively common event among pregnant women with severe dengue is preterm labor. Although there are not many reports in the literature to support the decision, most indications are towards inhibition of preterm labor.^(71)^ In these cases, the most appropriate drug for tocolysis is atosiban, an inhibitor of oxytocin. Terbutaline, magnesium sulfate and nifedipine have limitations for use in pregnant women with dengue, especially in the shock phase. Among them, nifedipine would be the one with the best profile to be used in pregnant women with dengue treated in maternity hospitals without access to atosiban. The use of non-steroidal anti-inflammatory drugs is contraindicated at any stage of dengue. Depending on the gestational age, the use of corticosteroids is permitted for prophylaxis of parenchymal hemorrhages in the fetus/neonate and to accelerate the production of surfactant substances. Betamethasone use and, alternatively, dexamethasone are recommended.

For pregnant women with severe preeclampsia or pregnant women with unsuccessful inhibition of preterm labor up to 32 weeks, magnesium sulfate offers proven maternal protection and perinatal neuroprotection, respectively. Although there are doubts about the safety of using magnesium sulfate in cases of pregnant women with dengue and risk of hemorrhage or shock, considering the risk versus benefit of its use in the clinical scenario of severe preeclampsia or the need for fetal neuroprotection, the use of this medicine is suggested. Logically, this pregnant woman should be in a hospital setting, with calcium gluconate immediately available.

Although controlled studies on fetal assessment in pregnant women with dengue are lacking, it seems reasonable that they have fetal vitality measured by electronic means such as ultrasound and cardiotocography. The frequency of these exams and which exam would be most appropriate will depend on the gestational age and the conditions of fetal well-being and its evolution. Aiming to resolve the pregnancy, fetal vitality must be assessed at the gestational age at which the community can offer support to the newborn. Although a gestational age greater than 28 weeks is often considered, it may be lower in scenarios with more neonatal infrastructure resources. The frequency and which test to use to assess fetal well-being will depend on the situation and the evolution of this well-being, a decision left to the judgment of the obstetrician. In general, cardiotocography should be performed daily and provides good results on fetal vitality. However, its high sensitivity may require supplementation with ultrasound to resolve doubts in some cases. Anyhow, ultrasound will be indicated every 3-5 days if the pregnant woman remains hospitalized. This exam provides important additional information about fetal vitality, other parameters of fetal physiology and development.

### Resolution of pregnancy

As a common premise in infectious syndromes with a marked inflammatory response, whenever possible, the resolution of pregnancy has better outcomes when carried out after the period of viremia and with maternal clinical improvement. Situations in which there is a need to terminate a pregnancy are infrequent, and occur in pregnant women with severe systemic involvement, requiring reflection on the impact of a surgical procedure and anesthesia on the evolution of the case. If the pregnancy is interrupted during a period of maternal decompensation and high viremia, it is also harmful for the newborn given the increased risk of hypoxia and vertical transmission. Clearly, in some situations, such as imminent spontaneous birth, there is no alternative management. The preparation for birth (spontaneous or elective birth), whenever possible, should include some additional care such as access to a bed in an advanced life support environment, blood and platelet reserves, a neonatologist in the room, experienced anesthetic and obstetric teams both to correct postpartum hemorrhage by using pharmacological measures (tranexamic acid, ergot derivatives, prostaglandins) and physical measures (uterine massage and intrauterine balloon), and to perform the surgical procedures needed if clinical measures to control possible hemorrhages were not effective.

Vaginal delivery is the best option for pregnant women with dengue, preferably with platelets above 50,000/mm.^(3,57)^ Due to the risk of maternal/perinatal obstetric trauma and hemorrhage, the use of forceps, vacuum extractor and episiotomy in parturient women with dengue must be carefully decided on.

Considering that the intramuscular route should be avoided for medication administration in patients with dengue in general, the universal postpartum prophylactic oxytocin should be administered intravenously as follows: 5 IU in bolus in slow infusion at every 3 minutes and, if necessary, until the 3^rd^ dose. Then, infuse another 20 IU in 500 ml for 2 hours (250ml/h) and another 20 IU in 500ml for 4 hours (125ml/h).^(72)^ In cases of cesarean section, special attention is recommended in strict surgical hemostasis in order to avoid intracavitary or abdominal wall bleeding and hematomas, as well as in postoperative analgesia, avoiding the use of non-steroidal anti-inflammatory drugs.

Objectively, the best option for pregnant women with dengue is to postpone non-urgent obstetric procedures (such as labor induction, uterine emptying maneuvers and cesarean section) until the patient’s clinical improvement.^(73)^

If there is a need to terminate a pregnancy, it is mandatory to ask about maternal conditions, fetal vitality, gestational age and neonatal survival prospects. If the use of corticosteroids and magnesium sulfate is indicated to improve postnatal survival conditions (protection against respiratory distress and cerebral hemorrhage), the use of these medications is permitted, taking care to measure maternal renal function and signs of shock before introducing magnesium sulfate.

As hemorrhage is one of the most serious complications of dengue during pregnancy and the puerperal period, the importance of active management for these women is reiterated, with controlled cord traction and intravenous prophylactic oxytocin. Chart 8 is a summary of the guidelines on obstetric care for pregnant women with dengue.

**Chart 8:** Obstetric management of pregnant women with dengue


Inhibition of preterm labor with aosiban (preferably) or nifedipine; the use of corticosteroids is allowed Magnesium sulfate can be used in pregnant women with dengue (without shock or bleeding) under 32 weeks or in cases of severe pre-eclampsia. Calcium gluconate should be available for immediate use if necessary Suspend use of ASA Suspend prophylactic anticoagulation in the presence of bleeding or platelets < 30,000 mm^3^ Avoid carrying out obstetric procedures that may be postponed In the case of unavoidable childbirth/cesarean section/abortion, the team must be prepared for a possible increase in bleeding, with blood reserves and prevention measures. Maintain platelets > 50,000/mm^3^ at birth and >70,000/mm^3^ at cesarean section Breastfeeding is allowed Monitor the newborn to assess vertical transmission if birth occurs between 10 days before and 10 hours after the onset of fever.

### Puerperal period

The spectrum of hemorrhages surrounds the puerperal woman with dengue in the immediate postpartum period, demanding careful evolution of the uterine involution and its contractility. The use of intravenous oxytocin for a longer period of time is permitted if needed.

The puerperal period is one that poses the highest risk of thrombosis in a woman’s life and in severe dengue, hemorrhagic events occur more frequently. Therefore, in the puerperal period of women with dengue there is a potential risk of these two complications. To minimize the risk of venous thromboembolism in patients able to walk, the use of compression stockings and encouragement of walking are recommended as prevention strategies. For bedridden puerperal women or with limited mobility, a physiotherapeutic approach, massage of the lower limbs and compressive or pneumatic stockings will be indicated.

The risk of DENV transmission through breastfeeding appears to be a rare event; only one case has been documented and described in the literature.^(42)^ Therefore, the recommendation is to maintain breastfeeding in puerperal women with dengue.^(43)^

For the newborn of a mother with dengue, the neonatologist must be alert in case follow-up is necessary because of the risk of vertical transmission of DENV and the development of dengue, especially when the maternal fever occurs between 10 days before and up to 10 hours after birth.^(19)^ Therefore, the puerperal woman’s discharge will depend not only on her clinical conditions, but also on the protocols of the neonatology service of each service.

The maternal health condition for “responsible hospital discharge” includes important variables as per information in chart 9.

**Chart 9:** Hospital discharge criteria for pregnant and puerperal women up to the 14th day postpartum (after dengue)


Hemodynamic stabilization for 48 hours Absence of fever for 24 hours Visible improvement in the clinical picture Normal and stable hematocrit for 24 hours Elevated platelets Assess social support

Source: Adapted from the Ministry of Health (2024).^(18)^

As there are no data in the literature to guide how long after giving birth a woman with dengue (without signs of alarm or severity) should be included in group A, we resort to the common sense that 14 days would be appropriate. This is the suggestion indicated in this Protocol.

The correct approach is to notify all pregnant or puerperal women with suspected dengue to the municipal manager using the “Dengue Notification Form”, and these women must receive the “Dengue Monitoring Card” in the initial consultation, which should be completed at each visit. The specific monitoring card for dengue has spaces for recording the care provided, information about warning signs, in addition to the scheduling of next appointments.

### Antenatal control after dengue

In most cases, the pregnant woman recovers from dengue and needs appropriate antenatal support to continue the pregnancy. The type of antenatal control provided to these pregnant women will depend on the severity of their illness. Cases considered mild with good clinical evolution and low inflammatory activity, can have low-risk follow-up.

Pregnant women who have had dengue with a prolonged evolution and slower recovery, showing visible organic impairment and a significant inflammatory response should be considered high risk prenatal care patients. These pregnant women often present placental impairment that could be translated in the future into increased rates of fetal growth restriction and oligohydramnios. In addition to the usual control, these pregnant women need ultrasound assessment of amniotic volume, vitality and fetal development, initially at monthly intervals. This period may be shorter and will depend on the evolution of fetal parameters at the judgment of the obstetrician. However, for pregnant women without access to this type of antenatal care and technological resources, measuring tape and fetal palpation can help detect possible deviations in fetal growth and amniotic fluid volume. If changes are detected, there is no other measure than referring the pregnant woman to specialized centers in accordance with the regional SUS flow.

Given the scarcity of data on the protocol management of pregnant and puerperal women with dengue, it is urgent to develop studies that can support the conduct recommended here, many of which derive from the common sense of the professionals involved and their experiences.

### Prevention

Undoubtedly, vaccines are powerful resources to prevent some infections and there is a great effort by the scientific community to discover a safe and effective vaccine against dengue.^(74)^ However, none of the available vaccines against DENV were released for use during pregnancy, as they contain attenuated viruses in their composition.^(14,75)^ Therefore, given the limited use of vaccines during the gestational period and the lack of antiviral treatment for dengue, the success in controlling this disease is based on general prophylactic measures.^(22,26)^

In primary prophylaxis, the highlights are attempts to control *Aedes aegypti* breeding sites, mechanical barriers preventing the mosquito from entering homes, use of insecticides, use of appropriate clothing and repellents.^(22)^ Considering the extermination of breeding sites, this control needs to occur in an expanded environmental manner. Until this happens, we recommend doing what we can correctly, eliminating breeding sites within homes and backyards.^(15,76)^

The use of insecticides by environmental vaporization (also called mosquito fogging or “*fumace*”) or at home, despite presenting some inconveniences and limitations, continues to be used in most municipalities in the country. Points for questioning its use include the harm to the environment, mosquito resistance to insecticides and the fact that it does not reach mosquitoes inside homes. Such topics generate varied opinions regarding its use.^(77)^

The use of fine mesh screens (metallic or plastic) on windows and doors to prevent *Aedes aegypti* from entering homes is recommended. The use of mosquito nets over the bed helps, although with limitations, as the *Aedes aegypti* mosquito has preferential daytime habits, combining with brightness and higher temperatures.^(26)^ Measures such as the use of insecticides or natural repellents in home environments are parallel strategies with variable and questionable percentage of effectiveness.

Traps that can attract, capture and contaminate mosquitoes with natural compounds or insecticides delivered in the form of nanoparticles are an important alternative, still in evolution.^(78)^ This approach can reach habitats not accessible by conventional means, acting at all phases of the mosquito’s life cycle (eggs, larvae, pupae and adult forms).^(79,80)^

Strategies for biological control of the *Aedes aegypti* population have shown promising,^(81)^ such as the use of the bacterium *Wolbachia pipiensis* with good results.^(82)^ The reproduction of *Wolbachia*-infected mosquitoes is affected; the eggs of non-infected females do not hatch when they are fertilized by a *Wolbachia*-infected male. In turn, if the female is already infected by this bacterium, her descendants will be resistant to DENV infection, in addition to causing a preponderant selection for females and death of males.^(83,84)^ Techniques that induce the selection of male mosquitoes already are used, still on a small scale and under strict quality control.^(59,85)^

Prophylaxis measures against *Aedes aegypti* bites are completed with the use of repellent on the entire exposed area of the skin. When wearing thin fabrics clothing, the product should be applied over the clothing. The most recommended repellents for use in pregnant women are based on “Icaridin”, “DEET” and “IR3535”.^(86)^ The intake of vitamins from the B complex, as well as repellents based on andiroba and citronella, among others, are ineffective as a repellent and their use is not recommended for this purpose.

The odor and the increase in CO_2_ exhaled by the pregnant woman’s body surface combined with the increase in her body temperature are important factors in attracting *Aedes aegypti*. However, these are difficult to control physiological variables. Another important variable is the color to which mosquitoes are attracted; they prefer darker colors. Therefore, wearing light-colored clothing that covers as much of the body surface as possible is recommended.^(87)^

As several of the procedures suggested in this Protocol are derived from expert opinions, we record the commitment of this group to reevaluate all the conduct reported here in light of new knowledge. The objective will always be to reduce maternal, embryonic, fetal and neonatal morbidity and mortality from DENV.
